# Detection of QTLs for seedling characteristics in barley (*Hordeum vulgare* L.) grown under hydroponic culture condition

**DOI:** 10.1186/s12863-017-0562-y

**Published:** 2017-11-07

**Authors:** Qifei Wang, Genlou Sun, Xifeng Ren, Jibin Wang, Binbin Du, Chengdao Li, Dongfa Sun

**Affiliations:** 1 College of Plant Science and Technology, Huazhong Agricultural University, Wuhan, 430070 China; 2 Biology Department, Saint Mary’s University, 923 Robie Street, Halifax, NS B3H 3C3 Canada; 3 Department of Agriculture & Food/Agricultural Research Western Australia, 3 Baron-Hay Court, South Perth, WA 6155 Australia; 4 Hubei Collaborative Innovation Center for Grain Industry, Jingzhou, Hubei 434025 China

**Keywords:** Barley (*Hordeum vulgare* L.), Seedling characteristics, Hydroponic culture, Quantitative trait loci (QTL), Dynamic QTL

## Abstract

**Background:**

Seedling characteristics play significant roles in the growth and development of barley (*Hordeum vulgare* L.), including stable stand establishment, water and nutrients uptake, biotic resistance and abiotic stresses, and can influence yield and quality. However, the genetic mechanisms underlying seedling characteristics in barley are largely unknown and little research has been done. In the present work, 21 seedling-related characteristics are assessed in a barley double haploid (DH) population, grown under hydroponic conditions. Of them, leaf age (LAG), shoot height (SH), maximum root length (MRL), main root number (MRN) and seedling fresh weight (SFW) were investigated at the 13th, 20th, 27th, and 34th day after germination. The objectives were to identify quantitative trait loci (QTLs) underlying these seedling characteristics using a high-density linkage map and to reveal the QTL expression pattern by comparing the QTLs among four different seedling growth stages.

**Results:**

A total of 70 QTLs were distributed over all chromosomes except 4H, and, individually, accounted for 5.01%–77.78% of phenotypic variation. Out of the 70 detected QTLs, 23 showed a major effect on 14 seedling-related characteristics. Ten co-localized chromosomal regions on 2H (five regions), 3H (two regions) and 7H (three regions) involved 39 QTLs (55.71%), each simultaneously influenced more than one trait. Meanwhile, 9 co-localized genomic regions involving 22 QTLs for five seedling characteristics (LAG, SH, MRL, MRN and SFW) at the 13th, 20th, 27th and 34th day-old seedling were common for two or more growth stages of seedling. QTL in the vicinity of *Vrs*1 locus on chromosome 2H with the favorable alleles from Huadamai 6 was found to have the largest main effects on multiple seedling-related traits.

**Conclusions:**

Six QTL cluster regions associated with 16 seedling-related characteristics were observed on chromosome 2H, 3H and 7H. The majority of the 29 regions identified for five seedling characteristics were selectively expressed at different developmental stages. The genetic effects of 9 consecutive expression regions displayed different developmental influences at different developmental stages. These findings enhanced our understanding of a genetic basis underlying seedling characteristics in barley. Some QTLs detected here could be used for marker-assisted selection (MAS) in barley breeding.

**Electronic supplementary material:**

The online version of this article (10.1186/s12863-017-0562-y) contains supplementary material, which is available to authorized users.

## Background

As the earliest domesticated crop and the fourth most important cereal crop in the world, barley (*Hordeum vulgare* L.) is not only widely used as human food and animal feed, but is also an ideal model of genetic research because it is early maturing, diploid, self- fertilizing and has a short growth period [1–3]. However, its growth and production are also greatly affected by seedling characteristics. The seedling stage (from seedling emergence to jointing) is considered as the most critical stage for barley production, and plays significant role in barley growth and development, including stable stand establishment, water and nutrients uptake, biotic resistance and abiotic stresses, and can influence yield and quality [4, 5]. In fact, strong seedling vigor and rapid seedling growth are important breeding targets in barley, as well as in other crops [6–8]. Seedling vigor has been identified to be associated with salt tolerance [9], drought tolerance [10, 11], nutrient uptake [12], weed competitiveness [8] and yield [13, 14]. It is difficult to assess seedling characteristics because a majority of important seedling characteristics are complex, quantitative traits determined by an array of developmental processes, genetic and environmental factors. The genetic mechanisms controlling seedling characteristics in barley are poorly known and little research has been done in this regard. Genetic variation for seedling characteristics in barley varieties does exist, and dissecting the genetic and molecular basis of seedling characteristics is necessary for genetic improvement of barley cultivars to enhance seedling vigor.

The information on the genetic basis underlying seedling characteristics, especially for root characteristics, is limited because it is difficult to obtain reliable phenotypic data in a large number of seedlings and in a complex external environment. It is also difficult to continuously measure these traits from the same seedling due to destruction of the seedlings in field experiments [15–17]. Therefore, an alternative approach is to examine seedling characteristics under controlled conditions using hydroponic culture, which has several advantages over field and other conventional techniques, including: (i) ease of investigation of seedling characteristics (as compared with field experiment), (ii) exclusion of soil and environment interference to increase measurement repeatability, (iii) investigation of large numbers of lines in short period of time, and (iv) precise control of the concentration of nutrient concentration [18]. At present, this method has been widely used in rice [19–21], maize [15, 22, 23] and wheat [16, 24, 25], and many QTLs have been detected at various water and nutrient regimes. Some researchers have used this method to identify QTLs associated with salt tolerance [3, 26], waterlogging tolerance [27–29] and nitrogen stress tolerance [30] in barley.

With the development of molecular markers and continuous encryption of genetic map, QTL analysis has become a powerful tool to dissect complex traits and identify chromosomal regions harboring genes that control these quantitative traits [31, 32], and has been extensively used for genetic dissection of important traits in barley [33–35]. The QTLs underlying yield [34, 36, 37] and quality [35, 38, 39] traits at later growth stages have been characterized previously, and the QTLs for seedling characteristics reported were mainly identified under salt tolerance [29, 40, 41], waterlogging tolerance [29, 42], drought tolerance [43] and nitrogen stress tolerance [30], while seedling characteristics related to grain yield and quality at early developmental stages are not well investigated.

The objectives of our study were to identify QTLs underlying seedling characteristics in hydroponics at seedling growth stages, and compare its QTL expression patterns among four different seedling growth stages.

## Results

### Evaluation of seedling characteristics

**Fig. 1:**
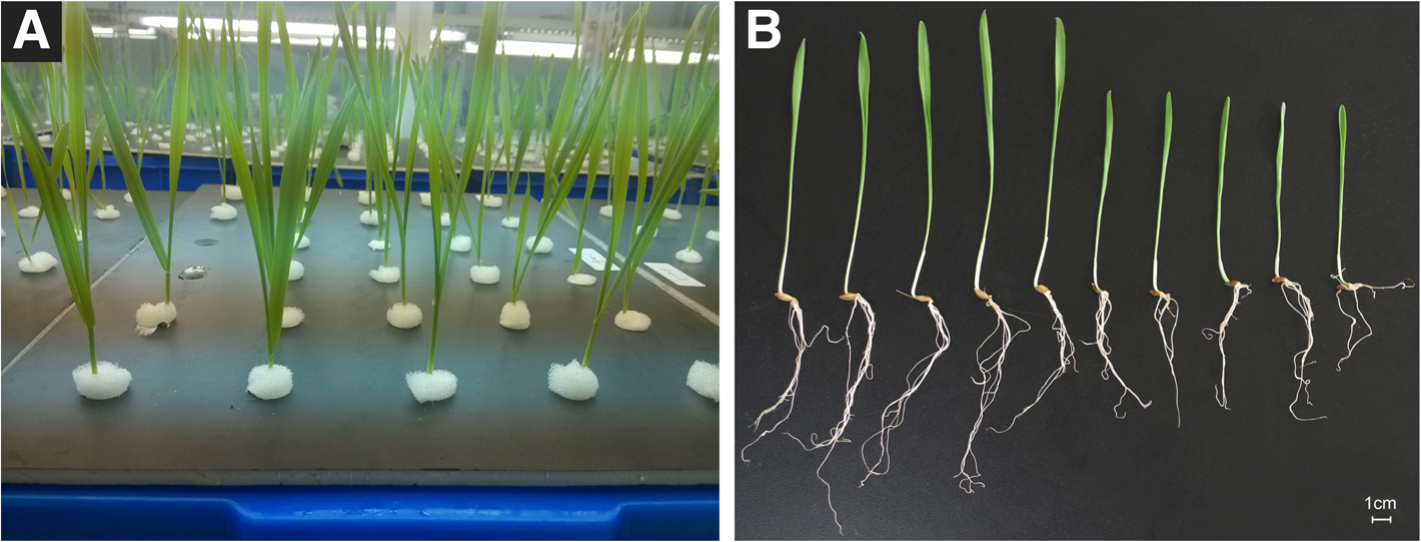
Seedling production using the hydroponic culture and parents seedling phenotyping. **a** The parents and doubled haploid (DH) population were cultivated in plastic tanks with eight liters of Hoagland’s nutrient solution. Throughout the experiments the roots were plunged into the nutrient solution. **b** Root systems and shoots of the parents Huadamai6 (from left 1–5) and Huaai11 (from left 6–10)

The DH population and two parents were grown hydroponically in a greenhouse as shown in Fig. 1a. Figure 1b showed phenotypic difference in the seedling of the Huadama 6 and Huaai 11. Huadama 6 and Huaai 11 have significant differences in seedling characteristics. All traits values, except FOLW, in Huadamai 6 were higher than those in Huaai 11. The T-test showed that two parents were significantly different on all traits (*p* < 0.05) but FILW and FOLW (Table 1).

**Table 1:** The statistics of the 122 lines from DH population and parents for the 21 seedling characteristics

Traits^a^	Stages^b^	Huadamai 6		Huaai 11		T value^d^	DH population							
		Mean	SD^c^	Mean	SD^c^		Max	Min	Mean	SD^c^	CV^e^	Skewness	Kurtosis	H^f^
LAG	S1	1.90	<0.01	1.67	0.27	2.15*	2.34	1.57	1.89	0.13	6.88	0.75	2.34	88.69
	S2	2.70	<0.01	2.45	0.10	5.00*	3.50	2.10	2.80	0.26	9.29	0.01	0.13	93.92
	S3	3.90	0.33	3.42	0.23	2.71*	5.30	3.05	3.93	0.40	10.18	0.59	0.81	95.89
	S4	5.55	0.23	4.94	0.22	4.42**	7.03	4.10	5.34	0.53	9.93	0.53	0.22	96.11
SH	S1	26.84	2.53	13.36	1.01	11.07**	26.40	12.10	18.86	3.22	17.07	0.22	−0.57	94.34
	S2	31.60	2.44	18.53	2.15	8.67**	33.60	13.23	25.41	3.94	15.51	−0.11	−0.28	93.23
	S3	33.82	0.71	22.83	1.75	14.07**	38.20	21.28	30.59	3.72	12.16	−0.03	−0.69	93.27
	S4	35.12	1.62	24.28	2.29	8.86**	42.17	24.23	33.08	4.43	13.39	−0.02	−0.72	94.53
MRL	S1	14.03	1.43	7.45	0.76	8.32**	14.76	5.47	10.05	2.04	20.30	0.25	−0.33	89.97
	S2	14.03	1.08	9.22	0.77	8.64**	19.68	7.50	11.67	2.39	20.48	0.90	0.87	91.39
	S3	14.80	0.63	11.18	1.42	5.59**	23.13	8.03	14.77	3.57	24.17	0.47	−0.56	89.95
	S4	15.53	0.43	11.40	1.29	6.69**	23.70	9.43	15.15	3.42	22.57	0.51	−0.47	89.86
MRN	S1	7.83	0.98	5.50	0.84	4.43**	10.20	5.20	6.88	0.93	13.52	1.21	1.83	92.90
	S2	10.00	1.67	5.83	0.98	5.26**	13.67	6.40	8.48	1.35	15.92	1.27	2.20	93.93
	S3	11.67	1.21	6.67	0.58	6.61**	18.33	7.40	11.12	2.13	19.15	1.08	1.61	94.82
	S4	14.00	1.22	11.00	1.41	3.42*	27.33	9.50	16.76	3.47	20.70	0.83	0.61	92.23
SFW	S1	0.57	0.05	0.24	0.03	11.81**	0.68	0.16	0.41	0.10	24.39	0.23	−0.26	94.94
	S2	0.95	0.09	0.44	0.08	8.84**	1.22	0.34	0.72	0.20	27.78	0.44	−0.45	93.69
	S3	1.36	0.16	0.64	0.11	6.88**	2.27	0.57	1.20	0.38	31.67	0.71	0.05	94.12
	S4	1.66	0.26	1.05	0.13	3.75**	3.81	0.88	1.94	0.67	34.54	0.60	−0.30	93.32
SLFW	S4	1.22	0.20	0.80	0.09	3.40*	2.87	0.64	1.44	0.50	34.72	0.69	−0.11	93.79
RFW	S4	0.44	0.07	0.25	0.04	4.39**	1.05	0.19	0.51	0.18	35.29	0.51	−0.21	91.79
SLDW	S4	0.17	0.03	0.11	0.01	4.80**	0.34	0.08	0.18	0.05	27.78	0.62	−0.20	91.30
RDW	S4	0.04	<0.01	0.02	<0.01	5.91**	0.08	0.02	0.04	0.01	25.00	0.45	−0.34	91.28
FILL		13.87	0.66	8.93	0.64	13.17**	14.50	6.80	10.85	1.73	15.94	−0.25	−0.65	97.05
SELL		25.94	1.65	14.28	0.76	12.97**	25.10	11.20	18.11	3.18	17.56	0.04	−0.51	93.07
THLL		26.63	2.00	17.44	1.94	7.70**	28.68	14.57	22.32	2.97	13.31	−0.06	−0.21	91.93
FOLL		27.94	1.49	19.17	2.46	6.43**	31.90	16.40	24.64	3.47	14.08	−0.14	−0.45	94.61
FILW		0.70	<0.01	0.68	0.04	1.00	0.87	0.53	0.74	0.07	9.46	−0.46	0.07	91.84
SELW		0.60	<0.01	0.44	0.05	6.53**	0.77	0.42	0.57	0.07	12.28	0.40	0.18	91.84
THLW		0.67	0.05	0.56	0.05	3.32**	0.96	0.44	0.64	0.09	14.06	0.36	0.52	91.76
FOLW		0.68	0.04	0.73	0.06	−1.53	1.08	0.50	0.76	0.12	15.79	0.29	−0.32	94.07
FILA		8.06	0.38	5.06	0.29	15.32**	9.37	3.86	6.63	1.26	19.00	−0.04	−0.64	95.64
SELA		12.92	0.82	5.35	0.92	13.06**	14.26	4.65	8.66	2.07	23.90	0.38	−0.44	92.68
THLA		14.74	1.54	8.16	1.55	7.03**	21.61	6.05	11.97	2.84	23.73	0.44	0.43	91.93
FOLA		16.23	0.87	11.63	1.29	6.12**	27.45	7.59	15.76	4.14	26.27	0.25	−0.21	94.82

^a^Trait abbreviations refer to Table 6

^b^S1 to S4 represented 13th, 20th, 27th and 34th day after germination, respectively

^c^ *SD* standard deviation

^d^*, **, significant at the probability level of 0.05 and 0.01

^e^ *CV* coefficient of variation in %

^f^ *H* heritability in %

The 21 seedling characteristics in DH population were also significantly different, with a highly phenotypic variation. The variation coefficient ranged from 6.88%–35.29%. Heritability ranged from 89.86% to 97.05% (Table 1). Frequency distribution histogram of the 21 seedling characteristics were shown in Additional file 1: Figure S1. Shapiro-Wilk test showed that some of the characteristics fitted the normal distribution, including LAG (S2), SH (S1-S4), MRL (S1), SFW (S1), LL (except FILL), LW and LA. In addition, transgressive segregation was observed for all 21 seedling characteristics.

### Detection of QTLs

**Fig. 2:**
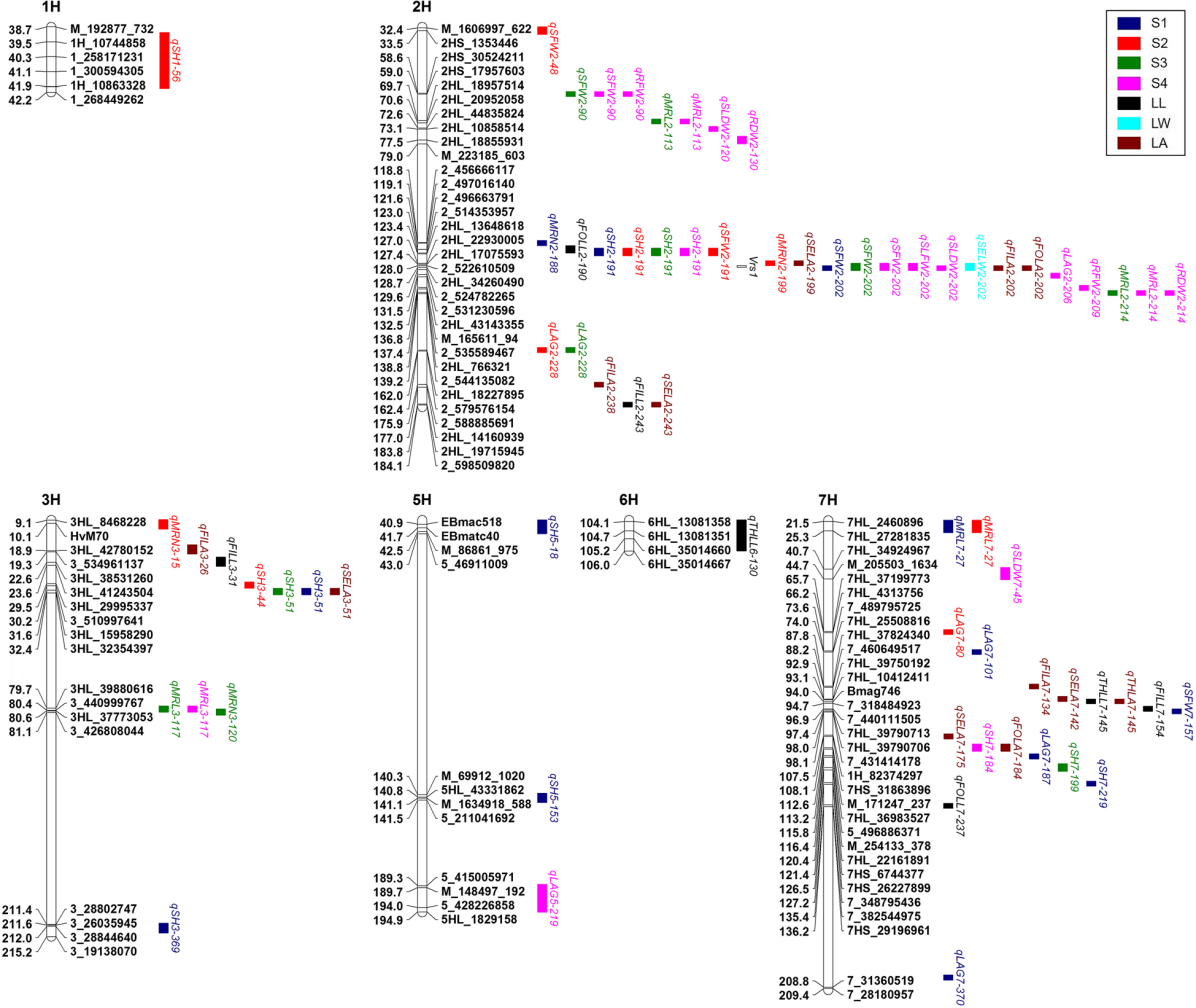
Chromosome locations of QTLs associated with 17 seedling characteristics detected in the Huaai 11 × Huadamai 6 DH population. QTL bars represented the 1.5-LOD support intervals from ICIM mapping. Genetic distance in centimorgans (cM) was placed at left. The row type trait was shown on linkage map (unfilled bars). Trait abbreviations refer to Table 6. S1 to S4 represented the 13th, 20th, 27th and 34th day after germination, respectively

**Fig. 3:**
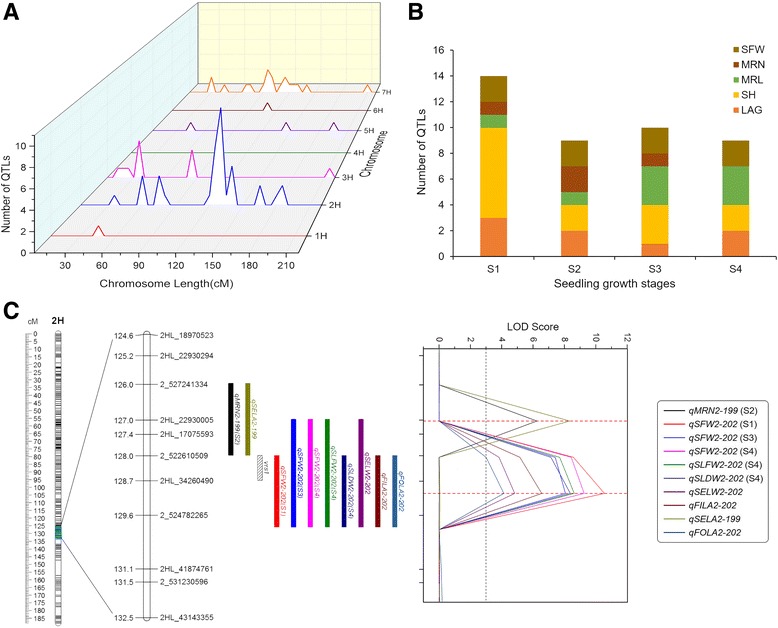
**a** Distribution characteristics of QTLs for 17 seedling characteristics in barley genome. **b** The number of QTLs for five seedling characteristics at each stage of seedling growth. Trait abbreviations refer to Table 1. **c** QTL-likelihood curves of LOD scores showed the locations of QTL for some seedling characteristics on chromosome 2H in the vicinity of *Vrs1* locus. Length of the rectangles corresponded to the 1.5-LOD support intervals based on the results of ICIM. *Vrs1* locus was indicated by hatched bars. The black dash line indicated that the LOD value was 3.0

A total of 70 QTLs on all barley chromosomes (except 4H) were detected in 17 of the 21 seedling characteristics using ICIM mapping (Table 2; Fig. 2). No QTL was identified for SELL, FILW, THLW and FOLW. Of these, a single QTL contributed between 5.01% and 77.78% to the phenotypic variance, with LOD value ranging from 3.04 to 27.07 (Table 2; Additional file 2: Figure S2). Most QTLs primarily dispersed on chromosome 2H (35 QTLs), 3H (11 QTLs) and 7H (19 QTLs) (Figs. 2 and 3a). Ten co-localized chromosomal regions affected more than one seedling characteristic distributed on chromosome 2H, 3H and 7H (Table 3).

**Table 2:** QTLs detected for 21 seedling characteristics under hydroponic culture conditions in the DH populations identified using inclusive composite interval mapping

Traits^a^	Stages^b^	QTLs	Chr.^c^	Pos.^d^	Left Marker^e^	Right Marker^e^	LOD	PVE^f^	Add^g^
LAG	S1	*qLAG7–101*	7	74	7_489795725	**7HL_25508816**	23.14	73.86	−0.11
	S1	*qLAG7–187*	7	116	**5_496886371**	M_254133_378	13.13	31.58	0.07
	S1	*qLAG7–370*	7	209	**7_31360519**	7_28180957	3.08	6.03	−0.03
	S2	*qLAG2–228*	2	162	**2HL_18227895**	2_579576154	10.31	26.1	0.13
	S2	*qLAG7–80*	7	66	7HL_37199773	**7HL_4313756**	5.26	12.51	−0.09
	S3	*qLAG2–228*	2	162	**2HL_18227895**	2_579576154	8.81	26.84	0.21
	S4	*qLAG2–206*	2	132	2_531230596	**2HL_43143355**	9.39	27.70	0.30
	S4	*qLAG5–219*	5	193	M_148497_192	**5_428226858**	3.17	8.39	0.15
SH	S1	*qSH1–56*	1	40	1H_10744858	**1_258171231**	4.53	7.50	−0.88
	S1	*qSH2–191*	2	123	**2_514353957**	2HL_13648618	10.14	18.61	1.53
	S1	*qSH3–51*	3	32	**3HL_15958290**	3HL_32354397	7.03	13.49	1.31
	S1	*qSH3–369*	3	212	3_26035945	**3_28844640**	3.41	5.49	0.75
	S1	*qSH5–18*	5	42	**Ebmatc40**	M_86861_975	12.54	24.40	1.67
	S1	*qSH5–153*	5	141	5HL_43331862	**M_1634918_588**	6.13	11.25	−1.12
	S1	*qSH7–219*	7	127	7HS_26227899	**7_348795436**	5.51	9.17	0.98
	S2	*qSH2–191*	2	123	**2_514353957**	2HL_13648618	10.54	17.74	1.83
	S2	*qSH3–44*	3	30	3HL_29995337	**3_510997641**	7.33	11.77	1.47
	S3	*qSH2–191*	2	123	**2_514353957**	2HL_13648618	9.07	19.06	1.79
	S3	*qSH3–51*	3	32	**3HL_15958290**	3HL_32354397	5.54	10.92	1.37
	S3	*qSH7–199*	7	121	7HL_22161891	**7HS_6744377**	8.30	17.43	1.56
	S4	*qSH2–191*	2	123	**2_514353957**	2HL_13648618	9.55	22.05	2.29
	S4	*qSH7–184*	7	113	M_171247_237	**7HL_36983527**	10.40	24.90	2.22
MRL	S1	*qMRL7–27*	7	25	7HL_2460896	**7HL_27281835**	3.69	13.16	0.75
	S2	*qMRL7–27*	7	25	7HL_2460896	**7HL_27281835**	4.04	12.92	0.88
	S3	*qMRL2–113*	2	70	**2HL_18957514**	2HL_20952058	4.09	10.58	−1.21
	S3	*qMRL2–214*	2	139	2HL_766321	**2_544135082**	6.06	16.23	1.54
	S3	*qMRL3–117*	3	80	**3HL_39880616**	3_440999767	4.58	12.65	−1.27
	S4	*qMRL2–113*	2	70	**2HL_18957514**	2HL_20952058	3.27	8.08	−1.01
	S4	*qMRL2–214*	2	139	2HL_766321	**2_544135082**	5.42	13.86	1.36
	S4	*qMRL3–117*	3	80	**3HL_39880616**	3_440999767	3.28	8.78	−1.02
MRN	S1	*qMRN2–188*	2	119	2_456666117	**2_497016140**	11.67	31.28	0.58
	S2	*qMRN3–15*	3	10	3HL_8468228	**HvM70**	3.34	7.71	0.26
	S2	*qMRN2–199*	2	127	**2HL_22930005**	2HL_17075593	6.25	19.08	0.66
	S3	*qMRN3–120*	3	81	3HL_37773053	**3_426808044**	3.22	11.53	0.73
SFW	S1	*qSFW2–202*	2	129	**2HL_34260490**	2_524782265	10.55	25.71	0.05
	S1	*qSFW7–157*	7	98	**7HL_39790706**	7_431414178	6.03	13.50	0.04
	S2	*qSFW2–48*	2	33	M_1606997_622	**2HS_1353446**	3.22	7.85	−0.06
	S2	*qSFW2–191*	2	123	**2_514353957**	2HL_13648618	11.70	32.39	0.12
	S3	*qSFW2–90*	2	59	2HS_30524211	**2HS_17957603**	3.04	9.30	−0.12
	S3	*qSFW2–202*	2	129	**2HL_34260490**	2_524782265	8.06	27.06	0.22
	S4	*qSFW2–90*	2	59	2HS_30524211	**2HS_17957603**	3.64	10.78	−0.22
	S4	*qSFW2–202*	2	129	**2HL_34260490**	2_524782265	9.26	30.50	0.40
SLFW	S4	*qSLFW2–202*	2	129	**2HL_34260490**	2_524782265	8.62	28.83	0.29
RFW	S4	*qRFW2–90*	2	59	2HS_30524211	**2HS_17957603**	3.60	9.81	−0.06
	S4	*qRFW2–209*	2	137	**M_165611_94**	2_535589467	10.15	32.06	0.11
SLDW	S4	*qSLDW2–120*	2	73	2HL_44835824	**2HL_10858514**	4.92	13.22	−0.02
	S4	*qSLDW2–202*	2	129	**2HL_34260490**	2_524782265	8.37	23.62	0.03
	S4	*qSLDW7–45*	7	41	**7HL_34924967**	M_205503_1634	3.16	8.17	−0.02
RDW	S4	*qRDW2–130*	2	78	**2HL_18855931**	M_223185_603	3.20	10.58	−0.01
	S4	*qRDW2–214*	2	139	2HL_766321	**2_544135082**	6.13	20.22	0.01
FILL		*qFILL2–243*	2	184	2HL_19715945	**2_598509820**	3.75	5.28	−0.40
		*qFILL3–31*	3	23	**3HL_38531260**	3HL_41243504	3.43	5.01	0.41
		*qFILL7–154*	7	97	**7_440111505**	7HL_39790713	22.87	48.43	1.21
THLL		*qTHLL6–130*	6	105	6HL_13081351	**6HL_35014660**	5.05	7.62	0.82
		*qTHLL7–145*	7	94	**Bmag746**	7_318484923	16.28	30.43	1.64
FOLL		*qFOLL2–190*	2	122	**2_496663791**	2_514353957	4.91	13.34	1.36
		*qFOLL7–237*	7	136	7_382544975	**7HS_29196961**	7.00	22.36	1.64
SELW		*qSELW2–202*	2	129	**2HL_34260490**	2_524782265	4.83	16.65	0.03
FILA		*qFILA2–202*	2	129	**2HL_34260490**	2_524782265	6.55	12.05	0.47
		*qFILA2–238*	2	176	**2_588885691**	2HL_14160939	5.48	9.94	−0.41
		*qFILA3–26*	3	19	**3HL_42780152**	3_534961137	3.17	5.56	0.31
		*qFILA7–134*	7	88	7HL_37824340	**7_460649517**	14.82	32.17	0.71
SELA		*qSELA2–199*	2	127	**2HL_22930005**	2HL_17075593	8.34	16.19	0.93
		*qSELA2–243*	2	184	2HL_19715945	**2_598509820**	4.10	7.41	−0.57
		*qSELA3–51*	3	32	**3HL_15958290**	3HL_32354397	3.20	5.98	0.56
		*qSELA7–142*	7	93	**7HL_39750192**	7HL_10412411	27.07	77.78	1.83
		*qSELA7–175*	7	108	1H_82374297	**7HS_31863896**	9.82	19.98	−0.93
THLA		*qTHLA7–145*	7	94	**Bmag746**	7_318484923	7.08	23.33	1.37
FOLA		*qFOLA2–202*	2	129	**2HL_34260490**	2_524782265	4.13	12.23	1.55
		*qFOLA7–184*	7	113	M_171247_237	**7HL_36983527**	5.27	16.75	1.71

^a^Trait abbreviations refer to Table 6

^b^S1 to S4 represented 13th, 20th, 27th and 34th day after germination, respectively

^c^Chromosome

^d^Genetic distance (in centimorgan) of each QTL from the top of the corresponding chromosome

^e^Markers in bold indicate the nearest ones linked to putative QTLs

^f^The phenotypic variation explained (in %) by each QTL

^g^Additive effect, positive values indicate that the alleles from Huadamai 6 increased trait values

**Table 3:** Putative pleiotropy or linkage of QTLs on linkage groups in barley

Chr.^a^	Pos.^b^	Left Marker^c^	Right Marker^c^	Involved traits^d^	LOD	PVE^e^	Add^f^
2H	59	2HS_30524211	**2HS_17957603**	SFW, RFW	3.04–3.60	9.30–10.78	–
2H	122/123	2_496663791	2HL_13648618	FOLL, SH, SFW	4.91–11.70	13.34–32.39	+
2H	127/129	2HL_22930005	2_524782265	MRN, SELA, SFW, SLFW, SLDW, SELW, FILA, FOLA	4.07–10.55	12.05–30.50	+
2H	137/139	M_165611_94	2_544135082	RFW, MRL, RDW	5.42–10.15	13.86–32.06	+
2H	184	2HL_19715945	**2_598509820**	FILL, SELA	3.75–4.10	5.28–7.41	–
3H	30/32	3HL_29995337	3HL_32354397	SH, SELA	3.20–7.33	5.98–13.49	+
3H	80/81	3HL_39880616	3_426808044	MRL, MRN	3.22–4.58	8.78–12.65	+/−
7H	93/94	7HL_39750192	7_318484923	SELA, THLL, THLA	7.08–27.07	23.33–77.78	+
7H	97/98	7_440111505	7_431414178	FILL, SFW	6.03–22.87	13.50–48.43	+
7H	113	M_171247_237	**7HL_36983527**	SH, FOLA	5.27–10.40	16.75–24.90	+

^a^Chromosome

^b^Genetic distance (in centimorgan) of each QTL from the top of the corresponding chromosome

^c^Markers in bold indicated the nearest ones linked to putative QTLs

^d^Trait abbreviations refer to Table 6

^e^The phenotypic variation explained (in %) by each QTL

^f^Additive effect; +, positive allele coming from Huadamai 6; −, positive allele coming from Huaai 11

**Fig. 4:**
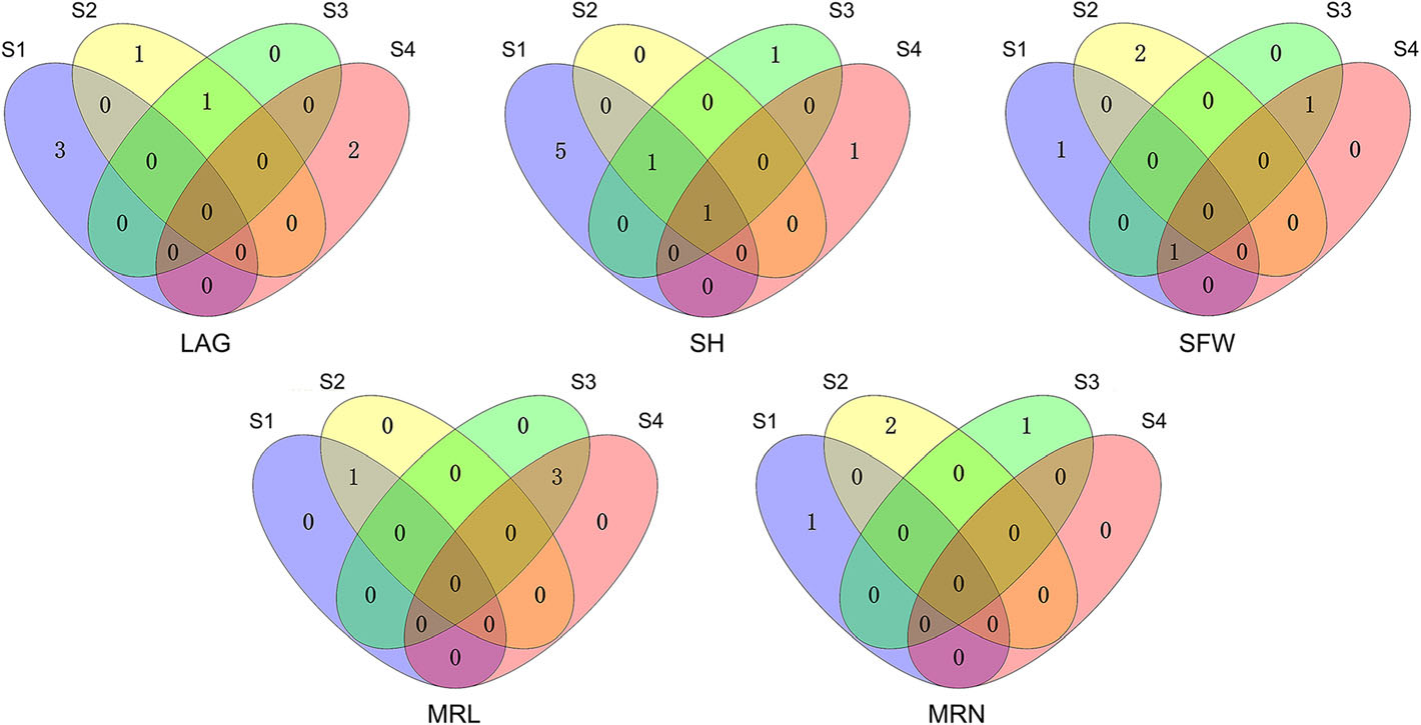
Venn diagram of QTLs detected for five seedling characteristics under hydroponic culture conditions at four developmental stages of seedling growth. Trait abbreviations refer to Table 6. S1 to S4 represented the 13th, 20th, 27th and 34th day after germination, respectively

**Fig. 5:**
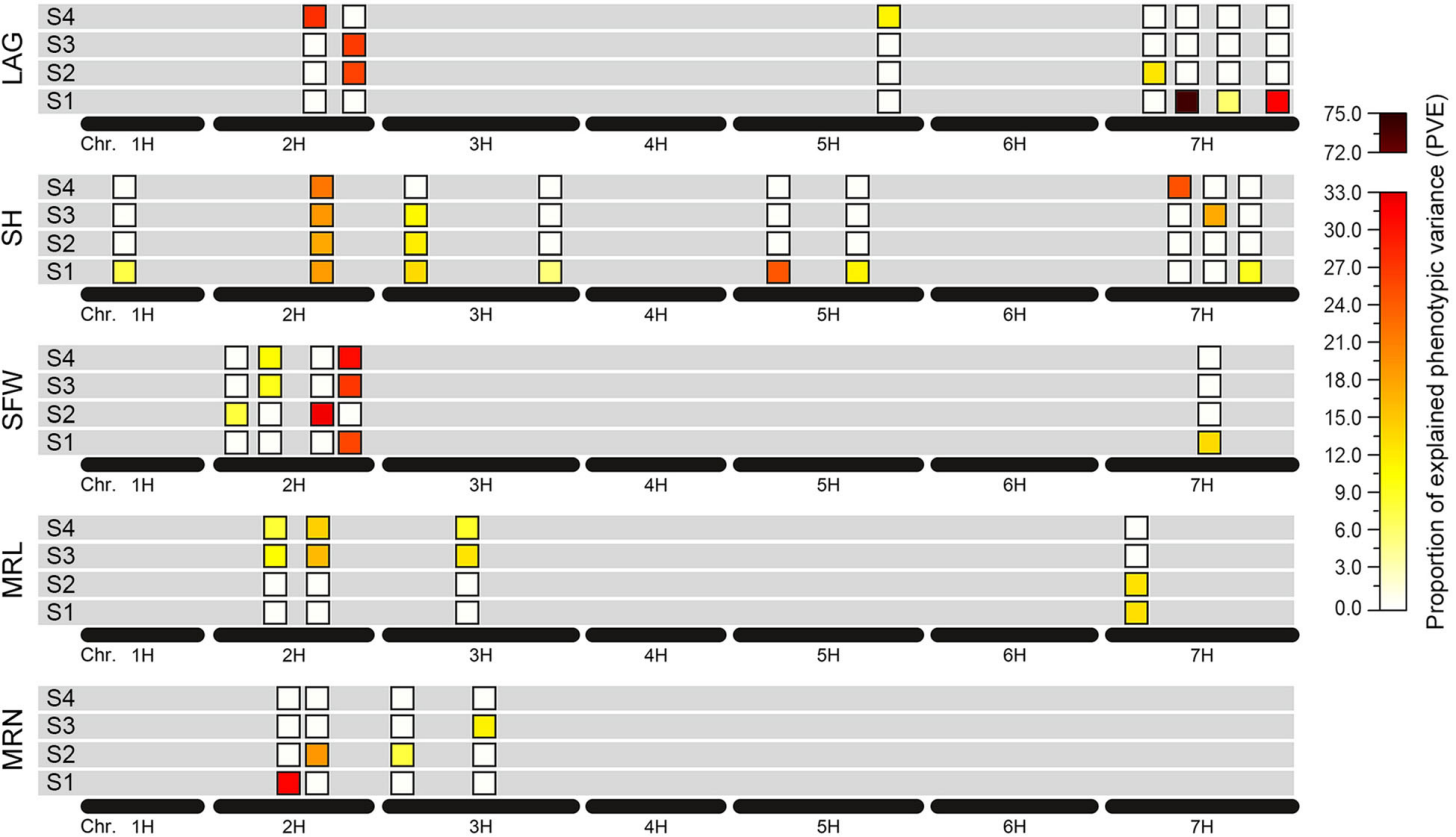
The temporal contributions of QTLs detected at any of the four stages to the proportion of explained phenotypic variation for five seedling characteristics. Trait abbreviations refer to Table 6. S1 to S4 represented the 13th, 20th, 27th and 34th day after germination, respectively

Dynamic QTL mapping on five seedling traits (LAG, SH, SFW, MRL and MRN) at four developmental stages identified 8 QTLs for LAG, 14 QTLs for SH, 8 QTLs for SFW, 8 QTLs for MRL and 4 QTLs for MRN (Table 2; Fig. 2). Phenotypic variation explained by single QTL ranged from 6.03% to 73.86% for LAG, from 5.49% to 24.90% for SH, from 7.85% to 32.39% for SFW, from 8.08% to 16.23% for MRL and from 7.71% to 31.28% for MRN. Interestingly, we found that only one QTL for SH was detected at all four stages, 8 QTLs for LAG, SH, SFW and MRL were detected for two or three stages, but also some QTLs were specific for either S1, S2, S3 and S4 (Table 4; Fig. 4). In addition, we also observed that some QTLs, such as the QTL at 123 cM on chromosome 2H, not only expressed at all stages for SH but also affected the SFW at the S2 stage. Similar QTLs were identified at 127/129 cM and 137/139 cM on chromosome 2H and at 30/32 cM and 80/81 cM on chromosome 3H. Two major QTLs for LAG (*qLAG2–228*) and SFW (*qSFW2–202*) explaining more than 20% of the phenotypic variation at more than one stage of seedling growth was identified on chromosome 2H. We analyzed the temporal-dynamics contribution of QTLs detected at any of the four stages to the proportion of explained phenotypic variation for five seedling characteristics (Fig. 5). The results showed that the proportion of explained phenotypic variance of some QTLs remained on a stable level at all stages of seedling growth; whereas, others substantially changed among the four stages of seedling growth.

**Table 4:** QTLs detected at two or more different stages of seedling growth

Traits^a^	Chr.^b^	Pos.^c^	Left Marker^d^	Right Marker^d^	LOD				PVE^f^					Add^g^			
					S1^e^	S2	S3	S4	S1	S2	S3	S4	Mean	S1	S2	S3	S4
SH	2H	123	**2_514353957**	2HL_13648618	10.14	10.54	9.07	9.55	18.61	17.74	19.06	22.05	19.37	1.53	1.83	1.79	2.29
	3H	30/32	3HL_29995337	3HL_32354397	7.03	7.33	5.54		13.49	11.77	10.92		12.06	1.31	1.47	1.37	
SFW	2H	59	2HS_30524211	**2HS_17957603**			3.04	3.64			9.30	10.78	10.04			−0.12	−0.22
	2H	129	**2HL_34260490**	2_524782265	10.55		8.06	9.26	25.71		27.06	30.50	27.76	0.05		0.22	0.40
LAG	2H	162	**2HL_18227895**	2_579576154		10.31	8.81			26.10	26.84		26.47		0.13	0.21	
MRL	2H	70	**2HL_18957514**	2HL_20952058			4.09	3.27			10.58	8.08	9.33			−1.21	−1.01
	2H	139	2HL_766321	**2_544135082**			6.06	5.42			16.23	13.86	15.05			1.54	1.36
	3H	80	**3HL_39880616**	3_440999767			4.58	3.28			12.65	8.78	10.72			−1.27	−1.02
	7H	25	7HL_2460896	**7HL_27281835**	3.69	4.04			13.16	12.92			13.04	0.75	0.88		

^a^Trait abbreviations refer to Table 6

^b^Chromosome

^c^Genetic distance (in centimorgan) of each QTL from the top of the corresponding chromosome

^d^Markers in bold indicated the nearest ones linked to putative QTLs

^e^S1 to S4 represented 13th, 20th, 27th and 34th day after germination, respectively

^f^The phenotypic variation explained (in %) by each QTL

^g^Additive effect, positive values indicated that the alleles from Huadamai 6 increased trait values

We assessed four seedling characteristics (SLFW, RFW, SLDW and RDW) at S4 stage, and detected one QTL for SLFW, 2 QTLs for RFW, 3 QTLs for SLDW and 2 QTLs for RDW (Table 2; Fig. 2). A single QTL identified for these four seedling characteristics could individually explain 28.83% (SLFW), 9.81%–32.06% (RFW), 8.17%–23.62% (SLDW) and 10.58%–20.22% (RDW) of the phenotypic variation. These QTLs were almost invariably located on chromosome 2H except for QTL *qSLDW7–45*. The major QTL *qSLFW2–202* was detected for SLFW on chromosome 2H around 127/129 cM and co-located with QTLs for SFW at S1, S3 and S4 (*qSFW2–202*), MRN at S2 (*qMRN2–199*) and SLDW at S4 (*qSLDW2–202*). Another major QTL was identified on chromosome 2H around 137/139 cM and had effect on MRN at S3 and S4 (*qMRL2–214*), RFW at S4 (*qRFW2–209*) and RDW at S4 (*qRDW2–214*).

For eight seedling characteristics (FILL, THLL, FOLL, SELW, FILA, SELA, THLA and FOLA), a total of 21 QTLs on chromosome 2H (nine QTLs), chromosome 3H (three QTLs), chromosome 6H (one QTL) and on 7H (eight QTLs) were identified. The LOD value ranged from 3.17 to 27.07, and individual QTL accounted for 5.01%–77.78% of the phenotypic variation (Table 2; Additional file 2: Figure S2). Three, two, two, two, one, four, five, one and two QTLs were detected for FILL, THLL, FOLL, SELW, FILA, SELA, THLA and FOLA, respectively. Interestingly, we found that six major QTLs, *qFILL7–154, qTHLL7–145, qFOLL7–237, qFILA7–134, qSELA7–142* and *qTHLA7–145*, distributed on chromosome 7H, had positive alleles from Huadamai 6. In addition, we also observed a number of QTLs that affected multiple traits, such as 127/129 cM on chromosome 2H.

## Discussion

Seedling characteristics include stem-and-leaf and root systems. A good stem-and-leaf system in the seedling stage is the basis for seedlings growth potential of robust, strong plants that are resistant to stress. To sustain a strong seedling vigor and rapid seedling growth, seedling must produce abundant nutrition. The amount of nutrition production mainly depends on the first few leaves of the seedling [44]. In addition, growth and development of healthy plants mainly rely on the strong root system (long MRL, high RDW and enough MRN), which were closely related to final grain yield and quality [45]. Some studies indicated that MRL and MRN were one of the most desirable traits contributing to drought avoidance, and RDW was another important parameter for the roots associated with water use efficiency and long-term drought [46, 47]. Recent studies have also confirmed that most of the seedling traits, such as leaf area (LA), seedling height (SH), seedling fresh weight (FW), seedling dry weight (DW), shoot dry weight (SDW) and root dry weight (RDW), were closely and positively correlated with yield component traits [48]. Previous genetic research on barley were focused mainly on the later stage; thus, the genetic mechanism underlying seedling characteristics is still lacking. In this study, we assessed QTLs underlying 21 seedling characteristics (including five seedling characteristics assessed at four stages of seedling growth) under hydroponic culture conditions and compared the QTL expression pattern among four different seedling growth stages.

### Advantages of hydroponic culture

For larger populations, it is difficult to assess seedling characteristics, especially whole-root systems, and for multi-stage barley planted in soil. Hydroponics culture allowed us to assess the same seedling over multi-stages. Genes or QTLs expression under hydroponic conditions might be different from field trials. However, the expression of genes or QTLs is always affected by the environment, while complex environments and uneven fertilizing in the field trial will affect the genetic mechanism of seedling characteristics, and may not reflect the intrinsic genetic program of barley seedling characteristics [19]. Thus, stable and consistent environmental control is needed to assess seedling characteristics. Recent studies have shown that QTLs identified under hydroponics conditions corresponded to QTLs detected in field trials, and suggested that a hydroponic system is a fast and cost-effective method for early QTL detection and marker-assisted allelic selection [30]. Consequently, we established a hydroponic system with homogeneous-growth conditions to evaluate barley-seedling characteristics. Temperature, humidity and lighting conditions were controlled by the automatic control system, and the nutrient solution was renewed every seventh day to ensure adequate nutrition. QTLs identified in this environment could reflect the intrinsic genetic mechanisms underlying barley seedling characteristics.

### QTL for seedling characteristics

With the same DH population used in this study, Ren et al. [49–52], Liu et al. [33] and Wang et al. [34] identified certain QTLs for physiological and morphological traits of flag leaf and some agronomic and quality traits (Additional file 3: Figure S3). Similar to their study, most of the QTLs identified in this study were also located on chromosome 2H and 7H. We found that some QTLs for seedling characteristics were co-located with QTLs for yield or yield-related traits identified in previous studies. For example, a significant QTL *qSH2–191* for SH detected in all stages of seedling growth was close to the SNP marker 2_514353957, which is likely the same to the yield-related QTL *qSms2–7* and *qTgw2–1* reported by Wang et al. [34] (Table 2; Additional file 3: Figure S3). In addition, the co-localized QTLs for THLL, SELA and THLA were detected on chromosome 7H and are likely the same to *qRWC7–9* and *qSPD7–9* for physiological and morphological traits of flag leaf at the pre-filling stage reported by Liu et al. [33] (Table 2; Additional file 3: Figure S3). These results suggested that seedling characteristics and yield are related to a certain extent.

In previous studies, QTLs conferring seedling height (SH) [29, 30], root dry weight (RDW) [46, 53–55] and stem-and-leaf dry weight (SLDW) [30, 42, 46, 53, 55] were reported on seven linkage groups, while QTLs for seedling fresh weight (SFW), leaf age (LAG) and main root number (MRN) were rarely reported in barley. QTLs for root length were identified on chromosome 1H, 2H, 3H, 4H, 5H and 7H [46, 53–55], and QTLs conferring root fresh weight (RFW) were previously reported on chromosome 1H, 2H and 5H [29, 42]. Recently, Hoffmann et al. [30] analyzed QTL underlying number of leaves longer than 2 cm after 14 days and length of the youngest completely unfolded leaf after 14 days, and found six QTLs for leaf number on chromosome 2H, 4H, 6H and 7H, and nine QTLs for leaf length on all seven chromosomes in wild barley introgression lines (S42ILs). By using GrainGenes3.0 website (http://wheat.pw.usda.gov/GG3/) to compare the genetic markers with those results present in this study, we found that some QTLs identified in our study are likely same to the QTLs reported in the past research. Such as, *qSH3–44* for SH is likely the same to the QTL *QHei.S42IL-3H* reported by Hoffmann et al. [30]. Arifuzzaman et al. [46] reported that QTL *QSdw.S42.2H.c* for SLDW on chromosome 2H was linked with the marker bPb-8143, and since the bPb-8143 is near the morphological marker *Vrs1*, as inferred from GrainGenes3.0 (http://wheat.pw.usda.gov/GG3/), we suggested that a co-localized QTLs for SLDW, SFW, SLFW, MRN, FILA, SELW, SELA and FOLA identified in our study is likely the same to the QTL *QSdw.S42.2H.c.* This QTL may be an important locus for controlling stem-and-leaf related traits in barley. The SNP marker 2HL_34260490 may be useful for marker-assisted selection (MAS) in barley breeding.

In addition, some new QTLs were also detected in this study. QTL for SH (*qSH7–184*) on chromosome 7H and QTL for RFW (*qRFW2–90* and *qRFW2–209*) on chromosome 2H, are likely different from the QTL reported by Xu et al. [29]. The QTL for MRL (*qMRL2–113* and *qMRL3–117*), QTL for RDW (*qRDW2–130* and *qRDW2–214*) and QTL for MRN (*qMRN3–120*) are likely different from those QTLs reported previously [42, 46]. The QTL, *qMRL7–27* (S1 and S2), located on chromosome 7H, is different from the *QRl.S42IL-7H.a* and *QRl.S42IL-7H.b*, as reported by Hoffmann et al. [30]. The *qMRL2–214* (S3 and S4) for MRL, mapped together with *qRDW2–214* (S4) for RDW and *qRFW2–209* (S4) for RFW on chromosome 2H, are different from those QTLs on chromosome 2H, as reported previously [55, 56]. This region may be important for controlling seedling root traits in barley.

### Selective expression of QTLs

According to developmental genetics, different QTLs may have different expression dynamics during trait development [7, 57]. Previous studies focused mainly on late-growth stages, where analysis was limited to the performance of a trait at a fixed time or stage of ontogenesis [33, 34, 58]. In the present study, we assessed five seedling characteristics (LAG, SH, SFW, MRL and MRN) at four stages of seedling growth (13th, 20th, 27th and 34th day-after germination) to reveal the QTL expression pattern.

A total of 42 QTLs involved 29 regions were detected for these five traits at four stages of seedling growth (Table 2; Fig. 4). Although the number of QTL expressed in each stage was approximately the same, only 9 regions were continually expressed in two or more different stages of seedling growth (Table 4; Figs. 3b and 4). Of these, only one of the nine regions was persistently expressed at all stages (Table 4; Figs. 4 and 5). The majority of the 29 regions for these five seedling characteristics were selectively expressed at different developmental stages. This result is in agreement with other studies where different QTLs could be identified at different growth stages, where only a few co-localized QTLs were detected at all stages [7, 59, 60]. The regions that continually expressed at different growth stages might play an important role in the growth and development of these characteristics. However, these regions do not always produce the same effect at each stage but show different developmental influences in different seedling growth stages. For example, two co-localized regions for SH were identified on chromosome 2H and 3H; its contribution to the phenotypic variance undergoes temporal changes. On chromosome 2H, a co-localized region contribution to the phenotypic variance decreased from 18.61% at S1 to 17.74% at S2 and then gradually increased until S4 (22.05%). Differently, a co-localization region on chromosome 3H contribution to the phenotypic variance gradually decreased from 13.49% at S1 to 10.92% at S3 and disappeared at S4. Besides, the co-localized regions for MRL, SFW and LAG contribution to the phenotypic variance also showed similar temporal changes. Generally, our results revealed that the genetic architecture of the five seedling characteristics shows dynamic temporal changes during growth and development.

### Pleiotropy or linkage of QTLs

We found here that many QTLs controlling multiple seedling characteristics were located at the same or overlapping marker interval on some chromosomes. This implied an existence of pleiotropic QTL or tightly linked QTL in our study. Xu et al. [7] suggested that if two QTL peaks are located very close to each other, and the 1-LOD support intervals completely or mostly overlapped, these two QTLs would be regarded as a single QTL having pleiotropic effect. In the present study, we delimited 1.5-LOD support intervals to confirm whether these two QTLs controlling multiple seedling characteristics can be identified as the pleiotropic QTL or tightly linked QTL. Based on this assumption, we found ten regions on chromosomes 2H, 3H and 7H that showed pleiotropic or tightly linkage effects on different seedling characteristics (Table 3).

Five regions on chromosome 2H were observed to simultaneously affect multiple seedling characteristics. Of these, a significant region with pleiotropic or tightly linkage effects on SFW, SLFW, SLDW, MRN, SELW, FILA, SELA and FOLA was close to both the SNP marker, 2HL_34260490 and the morphological marker, *Vrs1* (Fig. 3C; Table 3). The *Vrs*1 locus (controlling row type) has a pleiotropic effect on multiple traits [34, 61–63]. For example, Marquez-Cedillo et al. [61] and Wang et al. [34] reported that the *Vrs*1 locus had a pleiotropic effect on thousand grain weight, spike length, plant height, spikelet number on main spike and grain number per plant. Furthermore, some malting quality traits, such as grain-protein-percentage, diastatic power and soluble/total protein ratio, were also coincident with the *Vrs*1 locus reported by Marquez-Cedillo et al. [62]. Sato et al. [63] suggested that the fusarium head blight (FHB) resistance QTL in *Vrs*1 locus reported in two-row × six-row crosses might be pleiotropic effect of *Vrs*1 locus. Our study also showed a possible pleiotropic effect of the *Vrs*1 locus and provided evidence to further support previous suggestions.

On chromosome 3H and 7H, we also found that some regions showed pleiotropic effects on different seedling characteristics (Table 3). A pleiotropic QTL or tightly linked QTL, detected between SNP marker 3HL_29995337 and 3HL_32354397 near the SSR marker Bmag13, was related to SH and SELA. Examining the 3H linkage maps of Xue et al. [64] and GrainGenes3.0 (http://wheat.pw.usda.gov/GG3/) revealed that the marker bPb-6504 is near to the marker Bmag13, indicating that this QTL is likely the same to the *qSPL3c* associated with spikes per line. In the previous studies using the same DH population, QTL for heading date (*Qhd3–13*), culm length (*Qcl3–13*), two internode length(*Qitw3–13*) and three internode length (*Qith3–13*), as reported by Ren et al. [49, 51], and QTLs for stomatal conductance (*qGs3–13*) and transpiration rate (*qTr3–13*) reported by Liu et al. [33] were also near this region (Additional file 3: Figure S3). A QTL located at 93/94 cM on chromosome 7H showed that pleiotropic or tightly linkage effects on THLL, SELA and THLA is likely the same to the QTL (*qRWC7–9*) for relative water content and QTL (*qSPD7–9*) for relative chlorophyll content, as reported by Liu et al. [33]. These results provided favorable support for pleiotropic QTL or tightly linked QTL identified in our study. Further, this pleiotropic QTL or tightly linked QTL may be used for marker-assisted selection (MAS) and genetic improvement of barley seedling characteristics in barley breeding.

The phenomenon of pleiotropic effect or linked for QTLs universally exists in various crop genomes. Pleiotropic effects of genetic loci are thought to play a critical role in evolution, reflecting functional and developmental relationships among phenotypes [65]. The application of linked QTL and pleiotropic QTL for breeding purposes should be carried out with care, especially if the pleiotropy and linkage are in negative direction [7]. For example, the Huadamai 6 alleles with the pleiotropic QTL or tightly linked QTL on chromosome 3H increased main roots number but decreased maximum root length (Table 3). However, a pleiotropic QTL with opposite effects on the different traits complicated its application in barley improvement.

### QTL clusters in the genome

In our study, we found that most of QTLs were located on chromosomes 2H, 3H and 7H (Figs. 2 and 3a). It is noteworthy that many QTLs gathered in the same chromosome regions, even for the unrelated traits (Table 5). For instance, one QTL cluster region for RDW, SLDW, MRL, SFW and RFW was mapped on chromosome 2H close to the marker GBM1218. Liu et al. [33] reported QTL clusters for physiological and morphological traits of flag leaf (net photosynthetic rate, stomatal conductance, flag leaf area, flag leaf length, flag leaf width, relative chlorophyll content and leaf nitrogen concentration) in the similar region. Similarly, Ren et al. [49] detected QTLs for heading date traits in this region. Wang et al. [34] also detected QTLs for spikelet number on main spike in this region. Considering all information here, we suggested that this region may be a credible region for cluster of QTLs.

**Table 5:** Putative QTL clusters on linkage groups in barley

Num.^a^	Chr.^b^	Involved QTLs^c^	Interval	Position (cM)^d^
C1	2H	LAG(S2, S3), FILA, FILL, SELA	2HL_18227895-2_598509820	173.0 ± 11.03
C2	2H	SH(S1, S2, S3, S4), SFW(S1, S2, S3, S4), LAG(S4), MRL(S3, S4), MRN(S1, S2), SLDW(S4), SLFW(S4), RFW(S4), RDW(S4), FOLL, SELW, FILA, SELA, FOLA	2_456666117-2_544135082	129.0 ± 10.19
C3	2H	RDW(S4), SLDW(S4), MRL(S3, S4), SFW(S3, S4), RFW(S4)	2HS_30524211-M_223185_603	68.8 ± 10.18
C4	3H	FILA, FILL, SH(S1, S2, S3), SELA	3HL_42780152-3HL_32354397	25.6 ± 6.74
C5	7H	FILA, SELA, THLL, THLA, FILL, SFW(S1)	7HL_37824340-7_431414178	92.9 ± 5.19
C6	7H	SELA, SH(S1, S3, S4), FOLA, LAG(S1)	1H_82374297-7_348795436	117.4 ± 9.83

^a^The number of QTL clusters

^b^Chromosome

^c^QTL was represented by the trait names, and the QTL detected by the corresponding stage were shown in brackets

^d^The first figure indicated the distance from the top of the corresponding chromosome, and the second indicated the interval length

The most important QTL cluster was detected on chromosome 2H close to the morphological marker *Vrs*1, which was clustered with 22 QTLs (31.43%) for LAG, SH, MRL, MRN, SFW, SLFW, SLDW, RFW, RDW, FOLL, SELW, FILA, SELA and FOLA with contribution of 12.05%–32.39% (Table 5; Fig. 2). Wang et al. [34] reported QTL clusters for some agronomic traits (spikelet number on main spike, grain number per spike, spikelet number per plant, grain number per plant, grain weight per plant and thousand grain weight) in this chromosome region. Hori et al. [66] detected QTLs for plant height, spike exsertion length and thousand-kernel weight in this region. In addition, QTLs for spike number, floret number, grain number and hundred grain weight were also detected in this region by Chutimanitsakun et al. [67]. QTLs for seedling traits in this region may be a result of the pleiotropic effect of the *Vrs*1 locus or expression of the closely linked genes for *Vrs*1.

Another significant QTL cluster underlying LAG, FILL, FILA and SELA was detected on chromosome 2H between SNP marker 2HL_18227895 and 2_598509820 (Table 5; Fig. 2). The effects from Huadamai 6 were positive for LAG, but negative for FILL, FILA and SELA. Castro et al. [68] detected QTLs for photoperiod response in this region. Wang et al. [34] also detected QTLs for length of main spike, spike density, thousand grain weight and grain weight per spike in this region, and suggested that the SNP marker 2_598509820 in this region could be used for marker-assisted selection. In addition, another three QTL clusters were obtained on chromosome 3H and 7H, all of which enhanced development of stem-and-leaf traits (Table 5; Fig. 2).

QTL clustering in barley was repeatedly reported in some studies [33, 34, 56, 69]. In our study, we demonstrated several significant QTL clusters of seedling characteristics in barley under hydroponic culture. The genetic mechanism for this widespread phenomenon may be a linkage of genes and of pleiotropic effect of a single QTL in the same genomic region [70, 71]. However, whether it is linkage or pleiotropy, further research using fine mapping and cloning of QTLs or genes are needed validation.

## Conclusions

In this research, a number of genomic regions containing many seedling-related QTLs involved in different traits and diverse seedling growth stages were detected. The two parents used for mapping population construction are different in row types (six-rowed dwarfing barley cultivar Huaai 11 and two-rowed barley cultivar Huadamai 6). We found that chromosome 2H close to the morphological marker *Vrs1* contained the most important QTL cluster regions controlling multiple seedling characteristics. Some QTLs detected in this research could be used as a potential target for marker-assisted selection (MAS) in barley breeding.

## Methods

### Plant materials and hydroponic culture experiments

The barley double haploid (DH) population used in the current study was derived by anther culture from a cross between Huaai 11 and Huadamai 6 in our research group. This population consisted of 122 DH lines, have been described in our previous studies [33, 34, 49–52]. In the present study, 122 lines and the two parental cultivars were evaluated at seedling stage using the hydroponic culture (Fig. 1a). The experiment was conducted in greenhouse during the year 2014–2015 at the College of Plant Science and Technology of Huazhong Agricultural University, Wuhan, China. The conditions of greenhouse were set for 25/15 °C during a 16/8 h light/dark cycle, a photon flux density of 300 μmol m^−2^ s^−1^ during the 16 h light period, and relative humidity of 65% for 24 h. Barley seeds of the two parents and 122 lines were surface sterilized for 15 min in a 5% solution of sodium hypochlorite, rinsed thoroughly with distilled water, soaked in deionised water for 2 h, and then germinated for 5 days on plastic board floating over the deionized water at room temperature. After 5 days, 6 uniform seedlings from each line were selected for transplanting into foam board (polystyrene) suspended in the plastic tanks (34 cm × 25 cm × 12 cm, length × width × height) with nutrient solution. Each seedling was anchored to holes 1.5 cm diameter in foam board with sterilized sponge. Each foam board had 5 rows of holes, and each row had 6 holes for a total of 30 seedlings per board. The distance between two seedlings was 3.6 cm (between rows) and 4.0 cm (within a row), respectively. Each tank contained eight liters of revised Hoagland’s nutrient solution [72], which was renewed every seventh day to prevent nutrient exhaustion. When renewed the nutrient solution, each tank moves randomly to ensure that same environmental conditions. The solution PH was adjusted to 6.5 using diluted NaOH and HCl before refreshing. The two parents and DH lines were grown in completely randomized design with six replicates. In each replication, one seedling was used for data collection.

### Phenotyping of seedling characteristics

The phenotypes measured included leaf age (LAG), seedling height (SH, cm), maximum root length (MRL, cm), main roots number (MRN) and seedling fresh weight (SWF, g) at four growth stages (13th, 20th, 27th and 34th days after germination) on the same six seedlings for each line. Root fresh weight (RFW, g), stem-and-leaf fresh weight (SLFW, g), root dry weight (RDW, g) and stem-and-leaf dry weight (SLDW, g) were only measured at the 34th day after germination. For convenience, we used S1, S2, S3 and S4 to represent the 13th, 20th, 27th and 34th day after germination, respectively. In addition, leaf length (LL, cm), leaf width (LW, cm) and leaf area (LA, cm^2^) were measured in four leaves according to the leaf order of seedling. LL was measured for first leaf length (FILL, cm), second leaf length (SELL, cm), third leaf length (THLL, cm) and fourth leaf length (FOLL, cm). LW was measured for first leaf width (FILW, cm), second leaf width (SELW, cm), third leaf width (THLW, cm) and fourth leaf width (FOLW, cm). LA as measured for first leaf area (FILA, cm^2^), second leaf area (SELA, cm^2^), third leaf area (THLA, cm^2^) and fourth leaf area (FOLA, cm^2^). The methods of measurement are listed in Table 6.

**Table 6:** List of 21 quantitative traits investigated in the hydroponic experiment

Abbr.	Traits	Measurement standard	Unit
LAG	Leaf age	Number of leaves grown with time^a^	
SH	Shoot height	Length of the seedling from basis to the tip of the longest leaf blade	cm
MRL	Maximum root length	Length of the longest root from crown to root tip	cm
MRN	Main root number	Number of primary root	
SFW	Seedling fresh weight	Seedling weight after dry surface water	g
SLFW	Stem-and-leaf fresh weight	Stem-and-leaf weight after dry surface water	g
RFW	Root fresh weight	Root weight after dry surface water	g
SLDW	Stem-and-leaf dry weight	Stem-and-leaf weight after 2 days of drying at 75 °C	g
RDW	Root dry weight	Root weight after 2 days of drying at 75 °C	g
LL^b^	Leaf length	Length of the completely unfolded leaf from leaf basis to tip	cm
LW^c^	Leaf width	Maximum width of the completely unfolded leaf	cm
LA^d^	Leaf area	LA = (LL × LW) × 0.83	cm^2^

^a^The roll-leaf length measurement standard is, if the length of roll-leaf less than 1/3 the length of next leaf was recorded as 0.1, between 1/3 and 1/2 the length of next leaf was recorded as 0.3, between 1/2 and 3/4 the length of next leaf was recorded as 0.5, between 3/4 and the length of next leaf was recorded as 0.7, longer than the length of next leaf was recorded as 0.9

^b^The leaf length was measured including the first leaf length (FILL), second leaf length (SELL), third leaf length (THLL) and fourth leaf length (FOLL)

^c^The leaf width was measured including the first leaf width (FILW), second leaf width (SELW), third leaf width (THLW) and fourth leaf width (FOLW)

^d^The leaf area was measured including the first leaf area (FILA), second leaf area (SELA), third leaf area (THLA) and fourth leaf area (FOLA)

### Statistical analysis

The mean phenotypic values of the 21 seedling characteristics obtained from hydroponic culture were subjected to statistical analysis. Normality of distribution was tested using the method of Shapiro-Wilk. The descriptive statistics analyses were performed using SPSS programs (IBM SPSS Statistics, Chicago, IL, USA, http://www.ibm.com/analytics/us/en/technology/spss). The heritability was estimated [73]. Frequency distribution and QTL-likelihood maps for the seedling characteristics were drawn using the Origin programs (OriginLab, Northampton, MA, USA, http://www.originlab.com). *P*-value higher than 0.05 was deemed as significance level.

### QTL analysis

The high-density genetic linkage map constructed by Ren et al. [50] for the ‘Huaai 11 × Huadamai 6’ population was used in the QTL analysis, which contains 1894 SNP markers and 68 SSR markers, covering all 7 chromosomes and spanning 1375.80 cM of the barley genome with an average marker distance of 0.7 cM. The estimates of QTL positions and effects were determined by inclusive composite interval mapping (ICIM) [74] using the software QTL IciMapping 4.1 [75]. The mapping method was selected ICIM-ADD (additive effects) in ‘BIP (QTL mapping in biparental populations)’ function to analyze each trait from a single environment. Significant LOD (likelihood-of-odd) threshold for declaring a QTL of each trait was determined by 1000 permutations test with a Type 1 error of 0.05 [76]. The scanning step size was set at 1.0 centimorgan (cM) and the probability in stepwise regression (PIN) was 0.001. The rule recommended by Liu et al. [33] was used to name the QTLs, which were mapped on linkage groups using the software MapChart 2.2 [77]. The QTLs whose percentage of phenotypic variation explained (PVE) exceeded 20%, were considered as major QTL, and otherwise minor QTL. If the 1.5-LOD support interval of two QTLs overlapped, these two QTL would be defined as co-localized QTLs. However, we independently counted each QTL to clearly specify the number of detected QTLs. Graingenes website (http://wheat.pw.usda.gov/GG3/) was used to compare the maker information.

## Additional files

Additional file 1: Figure S1. Frequency distribution of 21 seedling characteristics in DH population. *P* value of Shapiro-Wilk test for each stage was shown, the hypothesis of normal distribution was accepted when *P* > 0.05 (significant at *P* = 0.05), and the trend lines of the accepted normal distribution were shown. Trait abbreviations refer to the Table 6. S1 to S4 represented the 13th, 20th, 27th and 34th day after germination, respectively. (JPEG 5396 kb)

Additional file 2: Figure S2. QTL likelihood map for 17 seedling characteristics in the DH population grown under hydroponic culture conditions using inclusive composite interval mapping. Genetic maps (all chromosomes together) of barley linkage groups were shown in the abscissa and LOD scores of each trait in the ordinate. The significant LOD threshold was determined to be 3.0 by 1000 permutations test. Trait abbreviations refer to the Table 6. S1 to S4 represented the 13th, 20th, 27th and 34th day after germination, respectively. (JPEG 4454 kb)

Additional file 3: Figure S3. QTL locations for previous studies and current studies detected in the Huaai 11 × Huadamai 6 DH population. QTLs were mapped to chromosomes using peak position and nearest marker. (JPEG 4137 kb)

## Abbreviations

cM: Centimorgan
DH: Double haploid
FILA: First leaf area
FILL: First leaf length
FILW: First leaf width
FOLA: Fourth leaf area
FOLL: Fourth leaf length
FOLW: Fourth leaf width
ICIM: Inclusive composite interval mapping
LOD: Likelihood of odd
MAS: Marker assisted selection
QTL: Quantitative trait locus
S1: 13th day after germination
S2: 20th day after germination
S3: 27th day after germination
S4: 34th day after germination
SELA: Second leaf area
SELL: Second leaf length
SELW: Second leaf width
SNP: Single nucleotide polymorphism
SSR: Simple sequence repeat
THLA: Third leaf area
THLL: Third leaf length
THLW: Third leaf width
