# Supplementary figures and images for: Detection of QTLs for seedling characteristics in barley (Hordeum vulgare L.) grown under hydroponic culture condition
Source: BMC Genet. 2017 Nov 7;18:94. doi: 10.1186/s12863-017-0562-y (PMC5678765; doi:10.1186/s12863-017-0562-y)

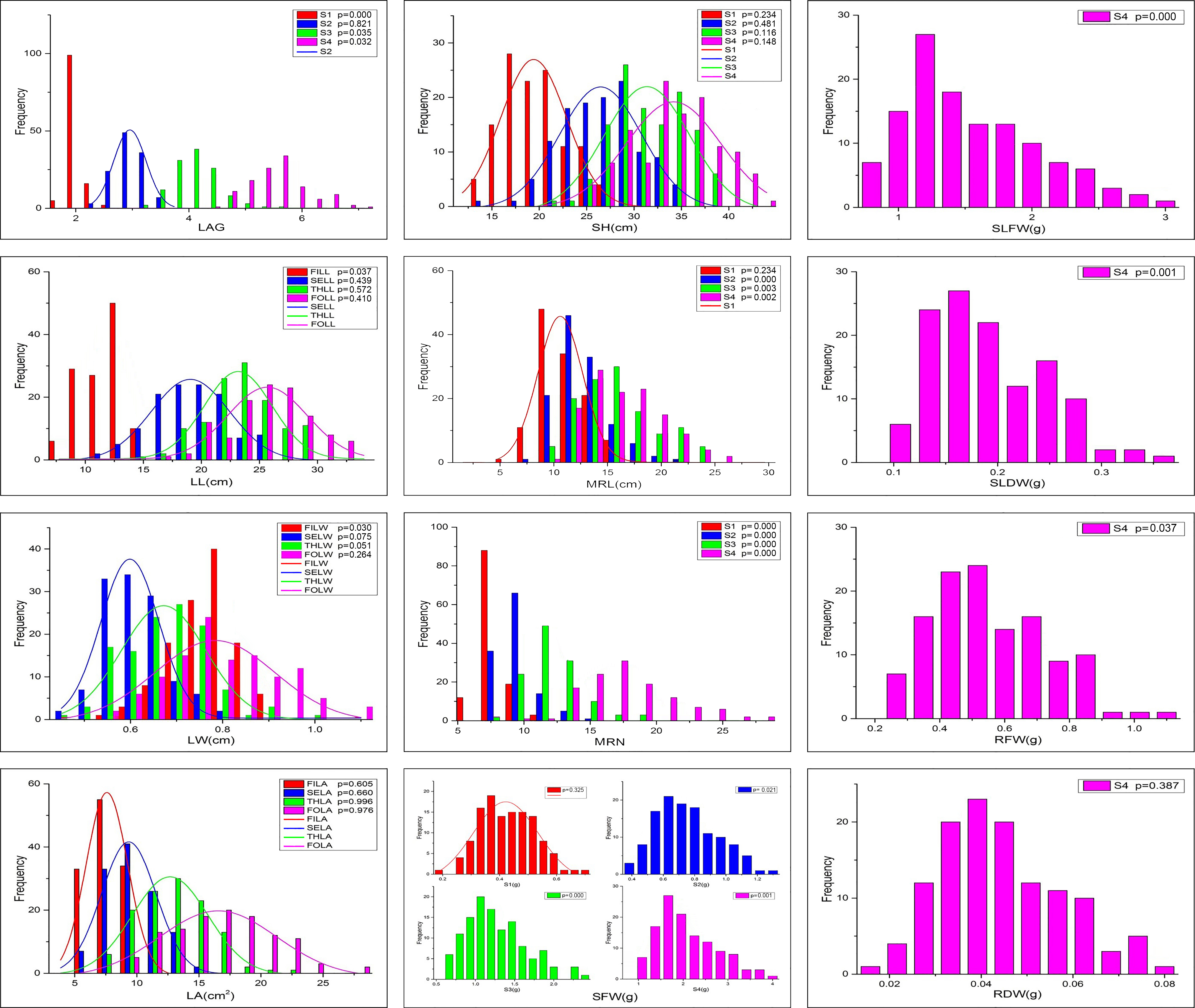

Supplement: Supplementary file 1 — Frequency distribution of 21 seedling characteristics in DH population. P value of Shapiro-Wilk test for each stage was shown, the hypothesis of normal distribution was accepted when P > 0.05 (significant at P = 0.05), and the trend lines of the accepted normal distribution were shown. Trait abbreviations refer to the Table 6. S1 to S4 represented the 13th, 20th, 27th and 34th day after germination, respectively. (JPEG 5396 kb) [file 12863_2017_562_MOESM1_ESM.jpg]

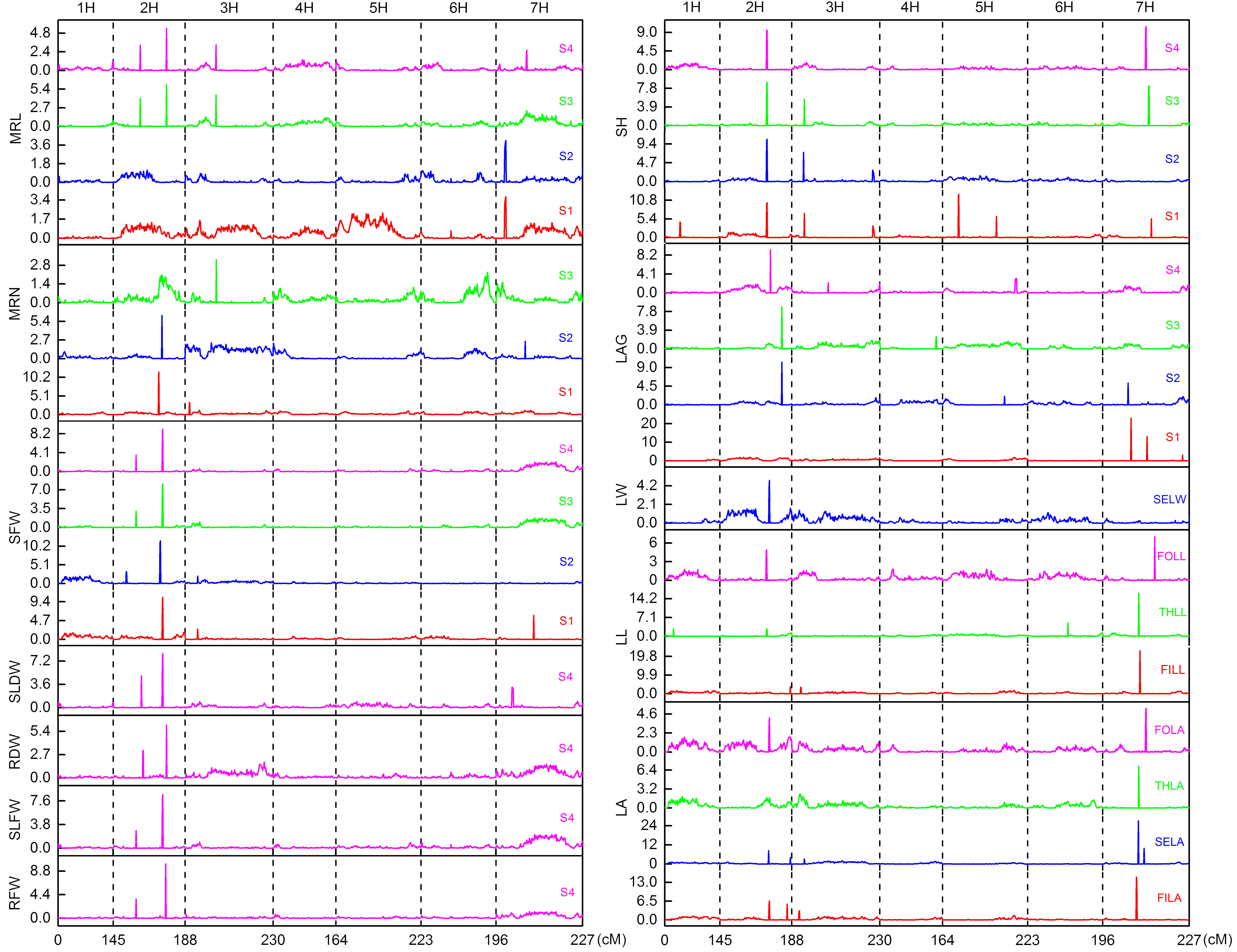

Supplement: Supplementary file 2 — QTL likelihood map for 17 seedling characteristics in the DH population grown under hydroponic culture conditions using inclusive composite interval mapping. Genetic maps (all chromosomes together) of barley linkage groups were shown in the abscissa and LOD scores of each trait in the ordinate. The significant LOD threshold was determined to be 3.0 by 1000 permutations test. Trait abbreviations refer to the Table 6. S1 to S4 represented the 13th, 20th, 27th and 34th day after germination, respectively. (JPEG 4454 kb) [file 12863_2017_562_MOESM2_ESM.jpg]

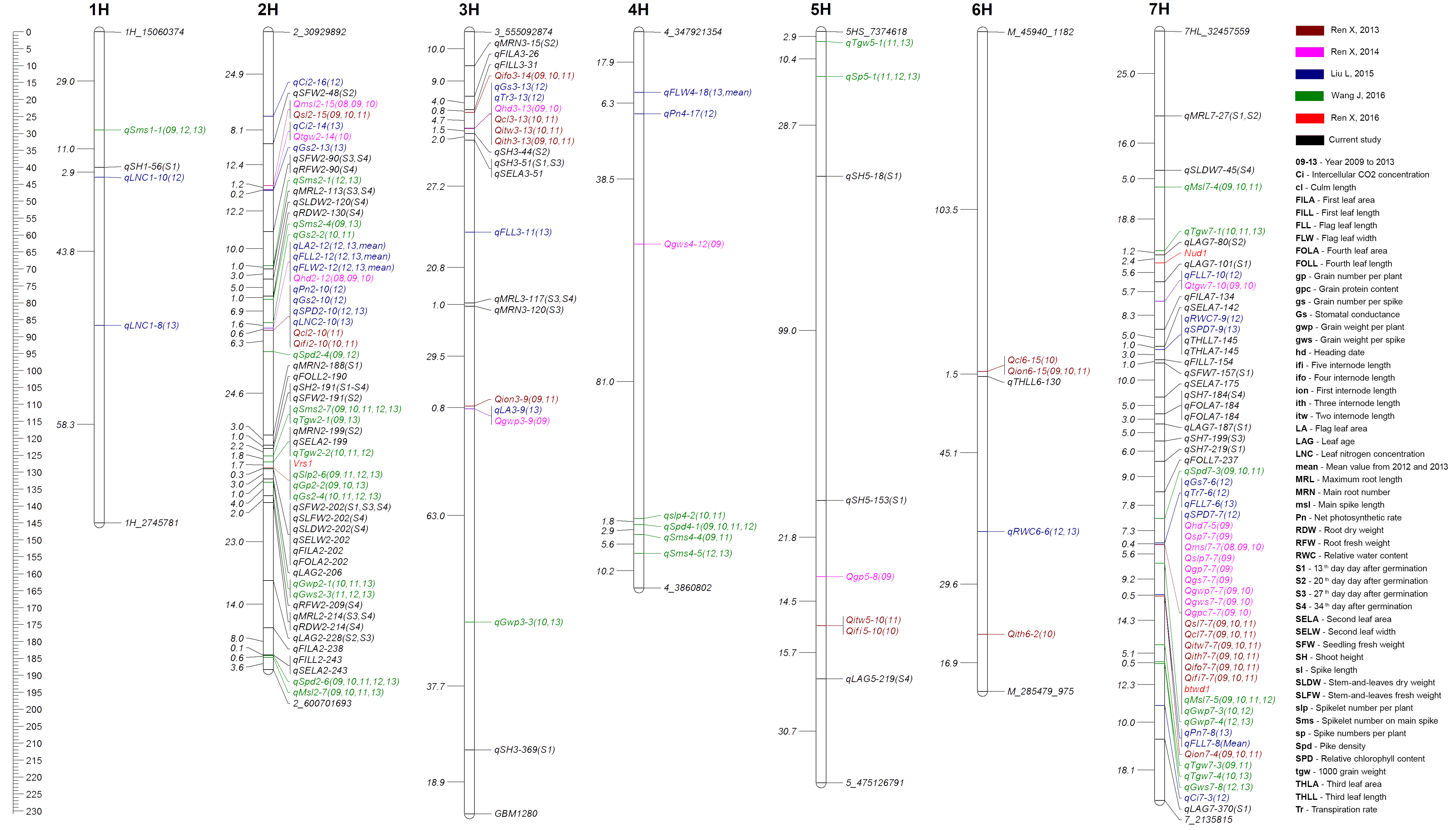

Supplement: Supplementary file 3 — QTL locations for previous studies and current studies detected in the Huaai 11 × Huadamai 6 DH population. QTLs were mapped to chromosomes using peak position and nearest marker. (JPEG 4137 kb) [file 12863_2017_562_MOESM3_ESM.jpg]
